# Databases for biomass and waste biorefinery – a mini-review and SWOT analysis

**DOI:** 10.1080/21655979.2023.2286722

**Published:** 2023-11-29

**Authors:** Morgen Mukamwi, Tosin Somorin, Raimonda Soloha, Elina Dace

**Affiliations:** a Chemical & Process Engineering, University of Strathclyde, Glasgow, Scotland, UK; b Institute of Microbiology and Biotechnology, University of Latvia, Riga, Latvia; c Department of Political Science, Riga Stradins University, Riga, Latvia; d Baltic Studies Centre, Riga, Baltic, Latvia

**Keywords:** Bioproducts, biowaste, circular bio-economy, biorefinery, feedstock, SWOT methodology

## Abstract

The world is facing problems of the increasing amount of resources wasted as the world population grows. Biowaste streams form a significant part of the overall waste generation, and a circular economy utilizing this biowaste will significantly reduce waste whilst lowering the anthropogenic carbon footprint. Due to their energy content and high concentration of hydrocarbon molecules, bio-based waste streams have the potential to be transformed into valorized products (energy, fuels, and chemicals) using biorefinery technologies. In this work, a mini-review has been conducted on available, mostly European databases on existing biomass types and biorefinery technologies to provide a framework for a desirable, comprehensive database connecting bio-based waste streams, biorefinery technologies and bioproducts, as well as the geographical distribution of feedstocks and biorefineries. The database assessment utilized the SWOT (strengths, weakness, opportunities, threats) methodology to support benchmark analysis and to identify critical gaps in underlying data structures that could be included in a single database. The results show that current databases are useful but insufficient for waste biorefineries due to limited quality and quantity as well as the usability of data. A comprehensive database or improved database cluster would be necessary, not only for technology development but for better investment and policy decisions. The development of the new database architecture would need to incorporate the aspects: expansion of database scope and content depth, improved usability, accessibility, applicability, update frequency, openness to new contributions, process descriptions and parameters, and technology readiness level.

## 1. Introduction

Global resource consumption is expected to double over the next 40 years [1] due to rapid urbanization and population growth. Projected annual waste generation is expected to reach 3.4 billion tonnes, an increase of nearly 70% over current generation rates [2]. In order to divert waste from landfills and reduce associated greenhouse gas emissions, global efforts are being made to minimize solid waste through waste design and the conversion of biomass and bio-based waste streams into value-added products. For the latter, the Bio-economy Action Plan [3] outlines actions in the EU to transform agricultural, urban, food and forest wastes into value-added products through the development of new sustainable biorefineries and efforts to replace fossil-based materials with bio-based, renewable and biodegradable alternatives. Other EU policy and action frameworks, such as the Circular Economy Action Plan [4], consider the use of waste materials to be essential because of the environmental benefits and opportunities they offer for reducing landfill and greenhouse gas emissions [5]. Therefore, the focus of this paper is on sustainable waste biorefineries and efforts to develop optimal combinations of feedstocks, process routes and products, and to understand product and process flows.

The International Energy Agency (IEA) Task 42 report on Bioenergy defines a biorefinery as the sustainable processing of biomass to produce a range of value-added products, including materials, feed, food, chemicals and energy (heat, power, fuel) [6]. This definition of a biorefinery allows it to be considered as a facility, cluster of facilities, processes or plants used in the conversion of biomass into products. There are different types of biorefinery operations, conversion technologies and process conditions [7], and ultimately a spectrum of products, taking into account different feedstocks and compositional variations. This diversity of biomass feedstock types and biorefinery technologies makes it difficult to select the optimal product. Furthermore, biomass types vary in different geographical regions and at different scales, meaning that some biorefinery technologies will be more suitable in one region than another. There is therefore a need to organize information into a more useful format by clearly linking feedstocks, biorefinery technologies and product chains, and by considering the geographical distribution of feedstocks, technologies and products. This compilation of information is best done in the form of a database.

Several databases have been created to provide information on different aspects of biomass waste and biorefinery technologies. Some of the databases focus on biomass and feedstock composition [8–10], biorefinery technologies [11,12], geographical distribution of feedstocks [8,13] and biorefinery installations [14–17] while others link feedstocks to biorefineries and/or products [8,18,19]. While a number of these databases are useful, the challenge is that a large number of databases exist primarily as ad hoc collections of limited information and often differ in purpose, scope and focus. The current databases are multiple sources for feedstock, biorefinery and product information, which makes it a lengthy process to retrieve information from these multiple sources, all with different accessibility properties. The data structure and the usability of databases for waste biorefineries are also not always clear.

In order to make waste biorefineries a reality, this paper fills the current gaps by reviewing existing databases and outlining the desirable features of an improved, comprehensive, single database containing the full depth of information on the entire feedstock-biorefinery technology-product chain. The database would have the necessary characteristics of usability, applicability, accessibility, update frequency, and scope. All of these require hierarchical data structures and appropriate tools, e.g. SWOT, for careful consideration.

The information from this study is essential as the development of a sustainable economy requires a significant integration of biorefinery concepts into the current value/supply chains. Such integration requires a good understanding of existing biomass and biorefinery processes and a good starting point would be a review of existing databases. The availability of biorefinery information in a consolidated database with desirable characteristics can inform policy makers to develop and implement relevant policies (such as subsidies, access to finance and use of biofuels) that support biorefinery operators and ensure that bioproducts are competitive. Investors would benefit from a consolidated biorefinery database by having comprehensive information in one place, enabling cost analysis and thus reducing investment in uncompetitive technologies or determining the market viability of different biorefinery products. Improved innovation through efficient policies can also help to reduce the processing costs of biorefineries, which are currently capital and energy intensive [20,21]. Finally, researchers will benefit as they seek ways to integrate biorefineries and improve existing or create new biomass conversion processes to produce a variety of value-added products.

This paper first presents the approach selected for database assessment and provides a classification of biomass feedstocks, biorefinery technologies and databases from a biorefinery perspective (Section 2). The databases identified in the literature are then reviewed and characterized according to the developed assessment framework (Section 3). The paper concludes with critical reflections on the potential use and further development of a comprehensive biorefinery database with the necessary features and information all in one database (Section 4).

## 2. Methodology

The methodological approach used in this study includes the following steps: i) review of electronic literature from the internet to establish a list of available databases and database classification, ii) SWOT analysis, and iii) data structure analysis. These steps are explained in the following sections.

### 2.1 Review of literature and database classification

**Figure 1.**
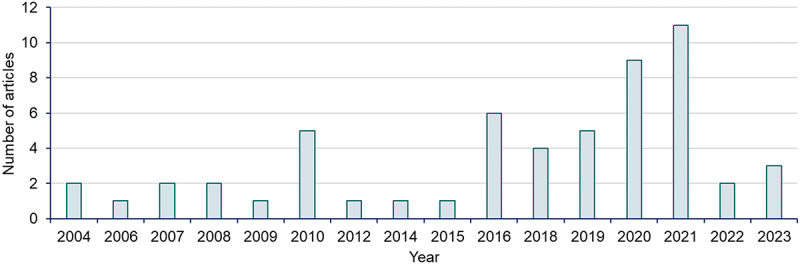
Publications on waste feedstock biorefinery conversion technologies and products reviewed in this paper.

The search was conducted using the following keywords: biorefinery database, biomass conversion technologies and biomass waste feedstocks, supplemented by a request to a network of researchers in the field of biomass and waste conversion technologies. The literature search was carried out using electronic resources on the internet available in Google Scholar, Microsoft Academic or google.com repositories. The use of Google Scholar was particularly beneficial as the repository provides an easy way to search and access a wide range of peer-reviewed scientific literature from many sources, such as journal articles, theses, academic publications or professional societies [22]. Microsoft Academic as a source of scholarly literature material is also advantageous in that it provides artificial intelligence-powered machine readers to process all the documents found by the Bing crawler to extract the relevant scholarly sources [23], thus improving the quality of results and speed of search. Figure 1 below is a graph showing the number of articles/sources used in this thesis against the years of publication. It is also important to note that the internet literature search for database information was carried out for databases mostly based in Europe, therefore this work is mostly anchored in a European context.

The databases reviewed were grouped into five categories according to their scope and emphasis: I) bio-based feedstocks including waste stream properties, II) biorefinery conversion technologies and processes, III) wiki/information register, IV-a) geographical distribution of feedstocks, IV-b) location of biorefinery installations, V) bio-based products and properties.

**Figure 2.**
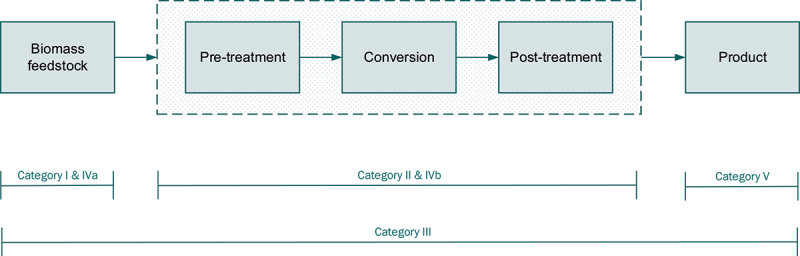
Schematic representation of database categorisation within the biorefinery concept.

Figure 2 illustrates the applicability of each database to the entire process chain from biomass feedstock to product. Thus, categories I and IVa cover biomass feedstock, categories II and IVb cover biorefinery technologies, while category III covers all stages of the biorefinery process chain from feedstock to product. The categorization of the databases was based on the main focus/theme of the database content, in the context that most databases have overlapping content between feedstock, biorefinery conversion process and bioproduct. Thus, category (I) databases focus primarily on feedstocks and their properties, category (II) on biorefinery technologies, category (III) without a specific focus but with approximately the same depth of information for each part of the whole feedstock-biorefinery-product chain, category (IV-a) on the geographical distribution of feedstocks, mostly in Europe, category (IV-b) on the existing locations of biorefinery facilities (mostly in Europe) and category (V) on bioproducts and their properties.

To the authors’ knowledge, there is currently no specific database dedicated to products derived from biomass feedstocks. However, if such a database becomes available in the future, it could be covered by a fifth category of databases. The diagram illustrates the full process of converting biomass into products in a biorefinery. Biomass goes through a pre-treatment step to condition it for optimal conversion. An example of a pre-treatment step would be drying wet biomass in preparation for combustion to generate heat in a biomass boiler. The post-treatment step conditions the product for final use, and an example is the separation of biomass residues from oils after a fast pyrolysis process. Within the four database categories (all except category V) a total of 19 databases were found, as shown in Table 1.

**Table 1.** Databases available in the literature.

No.	Category	Database name	Database content	Ref.
1	I	S2BIOM (I)	Biomass characteristics for lignocellulosic biomass	[8]
2	I	Phyllis2	Physico-chemical composition of (treated) lignocellulosic biomass, micro- and macroalgae, various feedstocks for biogas production and biochar	[9]
3	I	Refresh FoodWasteExplorer	Data on food waste streams	[10]
4	II	S2BIOM (IIa)	Biomass conversion technologies	[8]
5	II	S2BIOM (IIb), created as sub-database from Magic Match	Biomass conversion technologies Biomass feedstock matching with conversion technology and products	[8]
6	II	Charchive	Archive of (bio)char	[18]
7	II	POWER4BIO	Catalogue of bioeconomy solutions	[19]
8	III	Tech4Biowaste	“Wiki” (similar to Wikipedia) for biowaste technologies	[11]
9	III	TKI · BBE	Descriptions of biogenic conversion technologies	[12]
10	III	BT16 (Volume 2)*	Changes in the indicators of environmental sustainability (water quality, water quantity, air quality, greenhouse gas emissions, soil organic carbon, biodiversity) associated with selected production scenarios in BT16 (Volume 1)	[24]
11	III	GREET model*	Life-cycle impacts (air pollutants and greenhouse gas emissions, water consumption) of biomass processes (fuels, products, vehicle technologies, and energy systems)	[25]
12	III	BEIOM*	An economy-wide perspective on the environmental and socio-economic impacts of specific biofuels/bioproducts or their portfolios	[26]
13	IV-a	S2BIOM (III)	Biomass supply (Europe) Biomass cost/supply (Europe) Biomass cost-supply (Imports to Europe) Logistical components Value chain sustainability	[8]
14	IV-a	ReSourcer	Norwegian bioresources	[13]
15	IV-a	BT16 (Volume 1)*	Biomass availability and cost for the United States of America	[27]
16	IV-b	DataM	Chemical and material biorefineries in the EU	[14]
17	IV-b	Pilots4U	Asset register of biorefineries across Europe	[15]
18	IV-b	BEST	Facilities for the production of advanced liquid and gaseous biofuels for transport	[16]
19	IV-b	EERE	Integrated biorefineries in the United States of America	[17]

*These sources have a structure different from that of a database. Furthermore, they are mostly of U.S. relevance and not European relevance. Thereof they are not further reviewed.

### 2.2. SWOT analysis

**Figure 3.**
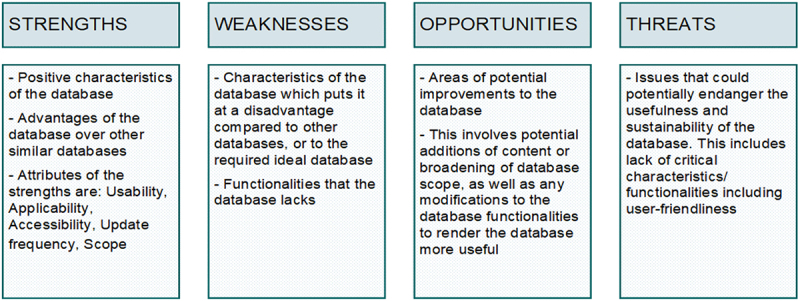
SWOT guideline for the database assessment.

A SWOT (Strengths, Weaknesses, Opportunities and Threats) analysis is used in this study to review and assess the databases with a view to developing a new, consolidated database containing the desired information in one place for biomass feedstocks, biorefinery technologies and bioproducts. As the name suggests, the SWOT tool explores the strategic relationships between an idea, concept or entity and its internal and external environment [28]. This approach has been widely used by other authors. Some examples of the application of SWOT analysis follow. Djellabi et al. [29] applied SWOT methodology to evaluate photocatalytic materials for use in large-scale environmental remediation. Ud Din et al. [30] applied the SWOT methodology to evaluate MOFs (metal-organic frameworks) for the synthesis of green methanol from H_2_ and CO_2._ Bonfante et al. [31] also used a SWOT analysis to compare current rare earth metal production routes and determine their alignment with the global sustainable transition. In the context of our study, strengths are the characteristics of the database that give it a relative advantage over other databases. The strengths of the database were assessed in several facets, namely usability, applicability, accessibility, update frequency and scope. Weaknesses are the characteristics of the database that make it a disadvantage compared to other databases. Opportunities are areas for improvement. Threats are those elements of the database that may threaten its competitiveness and sustainability of use. The guideline for this is summarized in Figure 3.

The definitions of the strengths presented in Figure 2 are described as follows:

- **Usability** - The ease with which a user can extract useful information from the database. This includes ease of navigation; sufficient metadata to aid the understanding of the data; necessary tools to manipulate the data e.g. filtering or sorting.
- **Applicability** - The purpose for which the database was created.
- **Accessibility** - The ability of the user to retrieve or access the data stored in the database.
- **Update frequency** - How often the database is updated including removal, amendment or addition of information in accordance with the latest scientific knowledge.
- **Scope** - The extent of the information included in the database. The scope includes biomass types, conversion technologies and product types.

### 2.3 Data structure analysis

In order to make a detailed assessment of the databases, it was necessary to stratify the biomass feedstocks, biorefinery technologies and biorefinery products into classes, analogous to the way in which the databases were classified. Stratification of data makes it easier to retrieve, analyze, track or manipulate the data [32]. This classification of data is particularly important for making the most of unstructured data from internet literature searches.

#### 2.3.1 Classification of biomass feedstocks

Biomass was classified into three categories as shown in Table 2 below. The categories (primary, secondary and tertiary) describe the source of the biomass or the stage at which the biomass becomes available for further use [33,34]. The second level classification stratifies the biomass according to its chemical composition. These classifications are useful in determining the focus of the databases. Due to the wide variety of biomass types, the biomass categories for the different databases were limited, but a desirable database would have all biomass classes in one database. Finally, the classification of the chemical composition is important as it directly determines the type of biorefinery technology that can be applied to a given product and thus the route for the utilization of the biomass [35].

**Table 2.** Classification of biomass feedstocks (elaborated from [33] and [34]).

No.	Category		Subcategory	Examples
1	**Primary biomass** Biomass is available “in the field” and needs to be collected to be available	1.1	Lignocellulosic biomass	- Virgin wood - Biomass from parks and gardens - Biomass from marginal areas
		1.2	Triglycerides	- Non-edible seeds - Oil energy crops
		1.3	Starchy biomass	- Starch energy crops
		1.4	Sugary biomass	- Sugar crops
		1.5	Others	- Algae - Seaweed - Manure
2	**Secondary biomass** formed during biomass processing	2.1	Lignocellulosic biomass	- Agro-industrial residue - Wood processing residue - Forestry residue - Residue from food processing - By-product from natural fibres processing- By-product from pulp and paper industry
		2.2	Triglycerides	- Waste vegetable oil from food industry - Animal fats
		2.3	Starchy biomass	- By-product from food processing
		2.4	Sugary biomass	- By-product from sugar crop processing
		2.5	Others	- Slaughter house waste - By-product from milk processing - Fish processing waste
3	**Tertiary biomass** Obtained after biomass-derived product has been used (waste or side stream)	3.1	Lignocellulosic biomass	- Waste construction wood - Wood packaging - Used natural fibres
		3.2	Triglycerides	- Waste cooking oil - Grease and fats
		3.3	Others	- Organic municipal solid waste - Sludges - Industrial and municipal wastewater

#### 2.3.2. Classification of biorefinery conversion technologies

There are different types of biorefinery conversion technologies. In this study, they have been classified into five categories: biochemical, chemical, thermochemical, physicochemical and other (processes not included in the first four categories). As shown in Table 3, this classification allows an objective comparison between the databases based on the reported biorefinery technologies. A desirable database would include all technologies in one database.

**Table 3.** Classification of biorefinery technologies.

	Category and definition		Technology	Technology description	Ref.
1.	**Biochemical Processes , [36]:** Biological treatment of biomass (use of enzymes and microbes) to produce value-added products.	1.1	Anaerobic digestion	The process through which micro-organisms break down organic matter in the absence of oxygen, to produce products such as biogas. Arrested anaerobic digestion (microbial carbohydrate digestion) also utilises organic-rich wastes to produce short-chain carboxylic acids (C_2_ to C_4_) acids such as acetic acid (C_2_), lactic acid (C_3_), propionic acid (C_3_), lactic acid (C_3_), succinic acid (C_4_) and butyric acid (C_4_), that can be used as fuels or chemicals.	,[36], [37]
		1.2	Composting	The biological process where micro-organisms convert organic matter into soil-like material termed compost.	[11]
		1.3	Enzymatic hydrolysis	The production of monomeric sugars from lignocellulosic biomass using specific enzymes (for example amylases, cellulases and hemicellulases) to break down the chemical bonds in cellulose and hemicellulose polymers.	[11]
		1.4	Fermentation	The use of micro-organisms to convert biomass feedstock (or biomass derived feedstock) into chemicals. There are three variations of fermentation: (1) Gas fermentation – the microbial conversion process that uses gaseous feedstock containing a mixture of carbon monoxide, carbon dioxide, methane and hydrogen to produce a specific product, such as fuels or chemicals. (2) Liquid fermentation – the fermentation that occurs in a liquid medium. (3) Solid state fermentation – this type of fermentation involves a low water content in the substrate. The solid substrate is inoculated with the microbe culture and the cultivation is mainly done under controlled conditions such as controlled temperature, light and humidity. Open-culture anaerobic (without oxygen) fermentation recovers medium-chain fatty acids (MCFAs – saturated monocarboxylic acids C_6_ to C_12_) from organic wastes, to produce aditives to animal feed or precursors for biofuel production.	[11], [38] [36] [39]
2.	**Chemical Processes [11]:** The use of chemicals to process biomass into products throughchemical reactions	2.1	Alkaline hydrolysis	This is a pre-treatment process which involves the removal of lignin from biomass using an alkaline medium, thereby improving the reactivity of remaining polysaccharides and decrystallisation of cellulose. Cellulose and part of hemicellulose remain in solid fraction after pre-treatment.	[8]
		2.2	Dilute acid hydrolysis	This is a pre-treatment of biomass to make it suitable for enzymatic hydrolysis. Dilute acid is added to the biomass feedstock and the mixture is held at elevated temperature for a short period of time. Hydrolysis of mainly hemicellulose occurs, releasing monomeric sugars and soluble oligomers from the cell wall matrix into the hydrolysate.	[8]
		2.3	Oleochemical processing	Processing of natural oils and fats, both of vegetable and animal origin, into numerous substances, such as fatty acids, biodiesel and glycerine.	[8]
3.	**Thermochemical Processes [30]:** Conversion of biomass feedstock to energy or chemicals using heat to drive chemical reactions.	3.1	Direct combustion of solid biomass	Thermochemical technique in which the biomass is burned in open air or in the presence of excess air, for heat or CHP.	[8], ][40]
		3.2	Fast pyrolysis	Biomass is rapidly heated to high temperatures in the absence of oxygen (O_2_) to produce mainly liquid bioproducts (bio-oil).	[41]
		3.3	Gasification	Thermochemical conversion of biomass into syngas.	[40]
		3.4	Torrefaction	Thermochemical process to reduce water and volatiles contents from the biomass, thus improving its fuel properties: energy density, hydrophobicity, elimination of biological activity, grindability and more homogeneous composition.	[11], [42]
		3.5	Treatment in subcritical water, or supercritical water oxidation	Organic materials are decomposed into smaller molecules through hydrolytic reactions.	[8], [43]
4.	**Physicochemical Processes [34]:** Conversion of biomass feedstocks to useful products through a combination of mechanical and chemical means.	4.1	Mechanical treatment	Physical (non-chemical) means of processing biomass, while the chemical structure of the biomass components is preserved. Some examples include addition of water to cellulose fibre material followed by pressing and drying into a panel, in the presence of pressure and heat, or the use of the whole biomass without the need for initial extraction of cellulose or certain components. Other processes utilise part of the biomass, mixing it with water and lime-based binder to produce pulp which can be used to create rigid construction materials.	[44]
		4.2	Extraction of fibres	Production of fibres from biomass.	[8], [][45]
		4.3	Extraction of protein	Extraction of protein from biomass without denaturation of the protein. Process involves cell disruption and pressing, filtration, adsorption and drying.	[8]
		4.4	Techniques from pulp and paper industry	The kraft process converts wood into wood pulp, which consists of almost pure cellulose fibres, the main component of paper. Lignin can be used in other biorefineries.	[8], [45]
5.	**Other processes**			Any other process that is not included in any other categories, for example, mushroom growing and insect farming.	[11]

#### 2.3.3. Classification of biorefinery products

Within the circular bio-economy, biorefinery products can be classified into six broad categories: food, feed, chemicals, materials, energy, and fuels, as shown in Table 4 [11,46. Ideally, all the product types should be listed in a single database. Classification of biorefinery products could be useful if the target product to be produced is already known. Then it would be useful to explore the possible biorefinery technologies and feedstocks that can be used to produce that product.

**Table 4.** Classification of biorefinery products [11],[46].

	Category	Examples of feedstock	Example of product
1	Food	Milk whey	Whey proteins (α-lactalbumin, β-lactoglobulin)
2	Feed	Rapeseed cake	High protein rapeseed meal
3	Chemicals	Waste cooking oil	Glycerol, Lactic acid
4	Materials	Lignocellulosic fiber	Fibres, Biocomposites
5	Energy	Lignocellulosic biomass	Heat, Electricity
6	Fuels	Food waste	Biogas, Ethanol

## 3. Results and discussion

A summary of the SWOT assessment of the databases is given in Table 5. A detailed description of the database evaluation is given in Table S1.

**Table 5.** SWOT summary of database assessment.

Database Category	STRENGTHS	WEAKNESSES	OPPORTUNITIES	THREATS
I − Biomass feedstock properties	**Usability** All databases are generally straightforward to navigate and use, with the necessary metadata provided to aid understanding. All databases provide links/functionalities for downloading and exporting content for offline use. **Accessibility** All databases are online and free to access. **Applicability** All databases are applicable to policymakers, research and industry **Update frequency** Databases Phyllis2 (Physico-chemical composition of (treated) lignocellulosic biomass) and Refresh FoodWasteExplorer (Data on food waste streams) are updated regularly whilst S2BIOM (I) (Biomass characteristics for lignocellulosic biomass) is a completed and closed project. **Scope** Databases S2BIOM (I) and Phyllis2 contain content on all 3 categories of biomass (primary, secondary and tertiary), although not all the chemical compositions of biomass are covered. Refresh FoodWasteExplorer only contains information on food waste (tertiary biomass) **Openness to new contributions** Databases Phyllis2 and Refresh FoodWasteExplorer are open to new content contributions, whilst database S2BIOM (I) is a completed and closed project hence no further updates could be expected.	Absence of regular updates on the closed project database S2BIOM (I) may render the information obsolete in the future due to the progressive advancement of the science of biorefinery technologies. The requirement to open an account before downloading Phyllis2 database content slows down information retrieval S2BIOM (I) uses units of measure that are not defined or explained, thus hindering full understanding of database content	Expansion of the scope to include the full range of biomass compositions would advance the use of all databases.	All databases risk a drop in level of usage and being superseded by other databases which would contain content on a wider scope of biomass compositions The requirement for opening an account impedes usage level of a database
II − Biorefinery technologies	**Usability** All databases except Charchive (Archive of (bio)char) are straightforward to navigate and use, with the necessary metadata. **Accessibility** While all the databases are online and free to access, only Charchive requires an account request to be made to the database host before accessing the database content. **Applicability** All databases except Charchive are applicable to researchers, policymakers, investors, governments. The stated aim for Charchive is for researchers to find and share information on different biochar materials **Update frequency** S2BIOM (IIa) (Biomass conversion technologies) was last updated 2017, S2BIOM (IIb) (Biomass conversion technologies and biomass feedstock matching with conversion technology and products) 2021, POWER4BIO (Catalogue of bioeconomy solutions) 2022, whilst no update information could be accessed for Charchive. **Scope** Most of the databases contain at least 10 of the 17 identified biorefinery technologies. S2BIOM (IIa) has 15, S2BIOM (IIb) has 10 whilst POWER4BIO has 10 technologies. It is not clear how many technologies Charchive database contains, as we did not have access to it. **Openness to new contributions** S2BIOM (IIa) and S2BIOM (IIb) are closed projects and hence not open to new contributions. POWER4BIO is open to content contributions, whilst no information on openness to contributions exists for Charchive database	Absence of regular updates on databases S2BIOM (IIa) and S2BIOM (IIb) potentially makes some of the content obsolete over time. Requirement for access approval for Charchive dissuades database use No download functionality on content for S2BIOM (IIa) and S2BIOM (IIb) reduce offline use of the databses Some of the biorefinery technologies do not have process descriptions in S2BIOM (IIa) None of the databases provide environmental impacts (and mitigation measures) of the reported technologies	Expansion of database scope to include all the 17 identified biorefinery technologies - Inclusion of technology readiness level (TRL) to show stage of development of the biorefinery technology	Lack of process description, access and regular updates reduce the usage frequency of the databases.
III − Biorefinery Wiki	**Usability** Databases have hierarchical menus and summary homepages which makes them easy to navigate through. **Accessibility** Databases are free to access. **Applicability** Applicable to researchers, policymakers, industry or other parties interested in the bioeconomy. **Update frequency** Tech4Biowaste (wiki for for biowaste technologies) database is currently a live project running from 2021 to 2023. TKI · BBE database (Descriptions of biogenic conversion technologies) was last updated in 2020. **Scope** Tech4Biowaste database contains 12 of the 17 biorefinery technologies, whilst TKI · BBE database contains 6 technologies. **Openness to new contributions** Tech4Biowaste database is open to contributions whilst TKI · BBE database is final and already a closed project.	The scope of the databases is somewhat limited even though the primary focus of these databases is biorefinery technologies. Some of the references from Tech4Biowaste database are from Wikipedia	Making the databases more comprehensive through expanding the biorefinery technologies covered	The use of some Wikipedia references in Tech4Biowaste database dissuade the use of this content by researchers Absence of continual update of the TKI · BBE database may result in some of the information becoming obsolete in the future.
IVa − Biomass feedstock geographical register	**Usability** Navigating through the databases is straightforward. **Accessibility** Databases are free to access **Applicability** Applicable to researchers, policymakers, industry or other parties interested in the bioeconomy. **Update frequency** S2BIOM (III) database was last updated in 2016. ReSourcer database does not have update frequency information. **Scope** S2BIOM (III) database (Biomass supply, cost, Logistical components, value chain sustainability) contains comprehensive content on biomass supply (Europe), biomass cost/supply (Europe), biomass cost-supply (Imports), logistical components and value chain sustainability. ReSourcer database (Norwegian bioresources) connects production companies that have leftover biological raw material with companies that can use these resources as an input in the production of new products. **Openness to new contributions** S2BIOM (III) is closed to contributions whilst ReSourcer is open to new contributions.	Lack of continual updates (S2BIOM (III)) to database information potentially makes some of the information obsolete ReSourcer database is in Norwegian language, thus some of the information can be lost in translation to English	There is a need for regular updates to database information Databases are most effective if information is provided in the language of the target audience	The lack of updates in S2BIOM (III) may limit the applicability of the database in the future ReSourcer database could benefit from a wider usage if a second version could be created in English
IVb − Biorefinery geographical register	**Usability** Navigating through the databases is straightforward, with drop down menus, filters, as well as process descriptions. **Accessibility** Databases are free to access. **Applicability** Applicable mostly to researchers, investors, as well as policymakers **Update frequency** In general, databases are updated regularly, though BEST (Facilities for the production of advanced liquid and gaseous biofuels for transport) and EERE (Integrated biorefineries in the United States of America) databases do not show last update date. **Scope** All databases show the distribution of various biorefineries across Europe, except EERE database which includes biorefineries located in the USA. **Openness to new contributions** All databases are open to new contributions of content	Lack of technology readiness level (TRL) as well as process descriptions on DataM (Chemical and material biorefineries in the EU) and Pilots4U (Asset register of biorefineries across Europe) databases Missing update information on BEST database EERE focuses on America only, and is thus mostly useful in America	Inclusion of TRL, process descriptions and database update information	Absence of TRL presents a challenge to policymakers as well as investors in making policy and investment decisions Lack of update information makes it difficult to establish the current state of the biorefineries.

The results show that none of the databases contain all the desired information, but rather are tailored to a specific content. For example, some of the databases were country specific, e.g. ReSourcer – Norway, EERE – USA, while others, e.g. S2BIOM (I, IIa, IIb, III) and DataM, covered wider geographical areas (Europe). The existing databases are essentially multiple isolated sources of information for different audiences, although there is some overlap between some of the databases. Retrieving information for the full chain from feedstock to biorefinery technology to product currently takes time due to the multiple sources that need to be consulted to get the full picture. It is therefore suggested that the new databases should target a wider audience (e.g. Europe). A wider audience allows for a greater depth of information and a greater number of new contributions to the database content, thus making the database more relevant. The depth of information also varies. For example, the S2BIOM (IIb) database (created as a Magic Match sub-database) does not contain process descriptions, whereas the POWER4BIO database does. The databases contain the same type of information (biorefinery technologies), but the depth (or volume) of the content is different. It would therefore be desirable for a database to have a greater scope and depth of content, as this would reduce the time required to retrieve and process information. Ideally, all the different types of information contained in all five database categories should be in one database, with clear links between one type of information and another. Consolidating information from different sources slows down the process of applying knowledge and making investment decisions.

One of the key pieces of information on biorefineries is the process description (as mentioned above), which helps to understand the whole process from biomass feedstock extraction to the final product. Within the databases, some of the biorefinery processes do not have a process description (such as in database S2BIOM (IIb)), or the process description is partial. S2BIOM (IIa) has partial process descriptions in the sense that some of the reported biorefinery technologies have not been provided with process descriptions. These information gaps would need to be addressed in the formulation of the desired database.

In general, a database should be designed to be easy to navigate, with the ability to download content to allow offline processing. Analysis of the results shows that some of the databases, such as S2BIOM (IIb) and Refresh FoodWasteExplorer, have a hierarchical menu design which is helpful in organizing data and linking data elements, but others, such as POWER4BIO, do not.

Accessibility is one of the most desirable features of a database. 14 of the 15 databases identified are freely accessible without the need to create an account. Only one database, Charchive, requires a request for an account and approval before access to the content can be granted. Accessibility is necessary to enable faster retrieval and processing of data for research, policy making and investment.

Biorefinery technology is constantly evolving and therefore one of the desirable features of a database is regular updating of the content. As shown in Table S1, 8 of the 15 databases have been updated within the last 5 years. Of the remaining databases, 3 were updated between 2016 and 2017 and 4 do not specify the frequency of updates. The more recent the update, the more useful and relevant the database is, as it would contain the latest developments in science. A feature related to the frequency of updates is the openness of the database to new content contributions from researchers or experts in the field. Contributions from database users are one of the fastest ways of updating the database content. However, this information would still need to be peer-reviewed before publication.

Biorefinery technologies are at different stages of development, and this is expressed as a technology readiness level (TRL). The TRL is a systematic assessment of a particular technology and a consistent comparison of the maturity of different types of technologies, all within the context of a specific operational environment [47][48]. A significant number of biorefinery technologies reported in the databases do not have TRL information (for example, 23 of the 74 processes described in the S2BIOM (IIa) database do not have a reported TRL, making it difficult to assess the commercial readiness of a given biorefinery process). This makes it difficult to assess the current commercial viability of biorefinery technology using a particular feedstock. The TRL status is an important part of the information needed by investors to make decisions. Therefore, the consolidated desirable database should provide TRL information for each biorefinery technology reported.

In order to accelerate the development of biowaste valorization technologies, it is essential to develop a single, comprehensive database with all the desirable features mentioned above, containing information on the entire feedstock-biorefinery technology-bio-product chain. This would speed up information retrieval, analysis and manipulation for faster technology implementation/development.

## 4. Conclusions and further outlook

An assessment of existing biomass and biorefinery databases helps to identify the necessary information that could be included in a single database. This would facilitate access to different types of information that would otherwise have to be found in two or more databases. A SWOT analysis was used to identify the advantages and disadvantages of existing databases and potential improvements in the design of a new database architecture. The frequency of database updates is crucial to ensure that the information available is up to date. Accessibility of the databases and the ability to extract data in a readable format (e.g. Excel file) are also important factors in ensuring that the information is accessible to a wide audience.

The content of the database itself, e.g. the level of detail of the information, affects the usability of the information. The inclusion of TRL would indicate whether the technology is at an early stage of research or is well developed and ready to be scaled up to an industrial level. Environmental impacts of biorefinery technology could help to assess the environmental sustainability of biorefineries. Overall, such information (e.g. through the application of life cycle analysis) is still lacking. Therefore, the development of the new database architecture would need to consider the following aspects: expansion of the scope and depth of the database, improved usability, accessibility, applicability, update frequency, openness to new contributions, process descriptions and parameters, and technology maturity. The inclusion of such information would make a database more comprehensive. However, a challenge could be the maintenance and verification of the information to ensure both the frequent updating of information and the validity of the information to be added. The database would be a consolidated database containing both technical and geographical information and would use the hierarchical menu structure to include all information from the identified database categories (feedstock, biorefinery technologies and products).

## Supplementary Material

Supplementary material_S1 Table.pdf

## Data Availability

The authors confirm that the data supporting the findings of this study are available within the article and its supplementary materials.
