# Supplementary material for: Databases for biomass and waste biorefinery – a mini-review and SWOT analysis
Source: Bioengineered. 2023 Nov 29;14(1):2286722. doi: 10.1080/21655979.2023.2286722 (PMC10761086; doi:10.1080/21655979.2023.2286722)
Supplement: Supplementary material_S1 Table.pdf [file KBIE_A_2286722_SM5294.pdf]

Supporting information

Databases for Biomass and Waste Biorefinery – A Mini-Review and SWOT Analysis

Morgen MUKAMWI[1], Tosin SOMORIN[1], Raimonda SOLOHA[2] , Elina DACE[2,3,*]

[1]University of Strathclyde, Glasgow, United Kingdom

[2] Institute of Microbiology and Biotechnology, University of Latvia, Riga, Latvia

[3]Department of Political Science, Riga Stradins University, Riga, Latvia

*Corresponding author

E-mail: elina.dace@lu.lv (ED)

S1 Table. Detailed SWOT Assessment of databases

CATEGORY I DATABASE: BIO-BASED FEEDSTOCK

No.	Database	Database content	Strengths						Weaknesses	Opportunities	Threats	Database URL
			**Usability**	**Accessibility**	**Applicability**	**Update frequency**	**Scope**	**Openness to new contributions**				
1	S2BIOM (I)	- Biomass characteristics for lignocellulosic biomass	- Database is easy to use and navigate.<br>- Database is in form of a pdf document containing biomass feedstock characteristics<br>- The metadata provided enables quicker understanding of the data<br>- The database has a download link to enable use of the information when offline<br>- Links to related databases are provided	- Free to access, with no requirement for setting up an account<br>- Database is online, hence accessible from anywhere within the set geographical regions	- Database purpose if to establish if certain biomass types can be used as feedstocks for specific biorefinery conversion technologies<br>- Database purpose is to assist industry, research and policy makers in their work.	- Last updated 1 November 2016	- Database covers lignocellulosic biomass in the primary, secondary and tertiary biomass categories	- This is a completed and closed project hence no further consideration for new content contributions	- There are no regular updates to the database content<br>- The database only covers lignocellulosic biomass and does not consider any other biomass types, hence it has limited scope<br>- Physical properties, proximate analysis and ultimate analysis are all lumped together in biomass properties, and it would be better to separate these into subsections to improve clarity	- The database can be made easier to navigate by converting it into a multilevel hierarchical menu design. The current pdf document results in more time being taken to scroll up/down or right/left to extract information<br>- Increasing the scope of types of biomass covered<br>- Inclusion of biomass subcategories such as triglycerides, starchy, sugary or other biomass.	- Since there is no room for new content or updates to existing content, the database risks being obsolete as science continues to advance<br>- The database may be superseded by other databases which include a more diverse range of biomass feedstocks, that is, inclusion of both lignocellulosic and non-lignocellulosic biomass feedstocks	https://s2biom.wenr.wur.nl/web/guest/conversion?p_p_id=conversions_WAR_Conversionsportlet&p_p_lifecycle=0&p_p_state=normal&p_p_mode=view&p_p_col_id=column-1&p_p_col_pos=1&p_p_col_count=2&_conversions_WAR_Conversionsportlet_cur=1&_conversions_WAR_Conversionsportlet_delta=75&_conversions_WAR_Conversionsportlet_keywords=&_conversions_WAR_Conversionsportlet_advancedSearch=false&_conversions_WAR_Conversionsportlet_andOperator=true&_conversions_WAR_Conversionsportlet_orderByCol=mainCat&_conversions_WAR_Conversionsportlet_orderByType=asc

2	Phyllis2	Physico-chemical composition of (treated) lignocellulosic biomass, micro- and macroalgae, various feedstocks for biogas production and biochar	- Database is easy to use and navigate as it has a hierarchical menu with self-explanatory navigation buttons<br>- Metadata is clear and enables easy understanding of the data<br>- Database contains download link for an Excel database, a useful functionality for data use in research	- Free access to database<br>- Online access to database	- Database is applicable for research, industry and policy makers	- Last updated 2022<br>- Database is updated and extended regularly<br>- Hence information contained is current and up to date	- Database contains information on lignocellulosic biomass composition, macro- and micro-algae, feedstocks for biogas production, biochar and torrefied biomass. The database provides data for individual materials or average values for a group of materials.<br>- Database contains information from all 3 biomass feedstock categories	- The database has functionality for downloading and uploading an Excel data template for adding more database content by users, although the data has to be within the scope of the database.	- Database requires one to open an account if one wishes to download database content. The requirement for opening an account slows down the process of downloading the required data for further use.<br>- Addition of information by users can only be done within the scope of the database.	- Expansion of the scope of the database to add other types of biomass not covered in the current scope	- The requirement for creation of an account to enable content download may discourage the use of the database	https://phyllis.nl/
3	Refresh FoodWasteExplorer	Data about food waste streams	- Database is relatively ease to use<br>- Database contains functionality to export the content in several file formats (html, xls, pdf)<br>- Filters can be applied to retrieve selected subsets of data, for example side stream (peel, stalks, seeds) and component groups (vitamins, minerals), and search results can be exported for offline analysis	- Free access to database<br>- Online access to database<br>- No requirement to register an account in order to be able to download database content	- Researchers, government agencies and industry including SMEs, and the general public	- Last updated 2021<br>- Database is updated and extended regularly	- Database contains food waste biomass and the properties of that waste<br>- Database contains information on the tertiary biomass category	- Database is open for new content contributions	- The units of measure are not defined, thus potentially hindering full understanding of database content	- Expansion of the database to include other biomass feedstocks which are not food waste	- The fact that the units of measure (especially units of measure that are not straightforward) may reduce the number of users of the database.<br>- The focus on only food waste may also reduce the number of users of the database, as users might prefer a database much more variety of content (food waste + non-food waste) in case they desire to build a biorefinery that can take both food waste and non-food waste biomass feedstock.	https://foodwasteexplorer.eu/

CATEGORY II DATABASE: BIOREFINERY TECHNOLOGIES

No.	Database	Database content	Strengths						Weaknesses	Opportunities	Threats	Database link
			Usability	Accessibility	Applicability	Update frequency	Scope	Openness to new contributions				
1	S2BIOM (IIa)	- Biomass conversion technologies	- Ease to use and navigate through database<br>- Comprehensive database<br>- Metadata provided aids understanding the database content<br>- Database layout clearly segregates different sections of the content, and the hyperlinks provided ensure a neat layout of the database that is easy to follow	- Free to access, with no requirement for setting up an account<br>- Database is online, hence accessible from anywhere	- Researchers, investors, government agencies, policymakers	- Last updated 2017	- Database contains 15 out of the 17 identified biorefinery technologies<br>- Process description present for most of the biorefinery technologies listed	- This is a completed and closed project hence no further consideration for new content contributions	- There are no regular updates to the database content<br>- Some of the biorefinery processes do not have process description or operating parameters listed<br>- Operating parameters for some technologies are lumped up within the process description<br>- No dowlonad link on database, making offline use of content difficult<br>- No environmental impacts provided for the biorefinery technologies	- Creation of an additional section to add operating parameters<br>- Inclusion of waste produced from processes, and waste handling methods<br>- Database could be expanded to include the other biorefinery technologies not included in this database	- Database information may become obsolete if not updated regularly	https://s2biom.wenr.wur.nl/web/guest/conversion?p_p_id=conversions_WAR_Conversionsportlet&p_p_lifecycle=0&p_p_state=normal&p_p_mode=view&p_p_col_id=column-1&p_p_col_pos=1&p_p_col_count=2&_conversions_WAR_Conversionsportlet_cur=1&_conversions_WAR_Conversionsportlet_delta=75&_conversions_WAR_Conversionsportlet_keywords=&_conversions_WAR_Conversionsportlet_advancedSearch=false&_conversions_WAR_Conversionsportlet_andOperator=true&_conversions_WAR_Conversionsportlet_orderByCol=mainCat&_conversions_WAR_Conversionsportlet_orderByType=asc

2	S2BIOM (IIb), created as sub-database Magic Match	- Biomass conversion technologies<br>- Biomass feedstock matching with conversion technology and products	- Easy to navigate through the database<br>- Links to related databases are provided	- Free to access<br>- Accessible online	- Researchers, investors, governments, policymakers	- Last updated 2021	- Optimal match between biomass crops and conversion technologies<br>- Database contains 10 of the 17 biorefinery technologies identified in this work	- This is a completed and closed project hence no further consideration for new content contributions	- Database webpage does not provide a link to the instructions on how to use the database<br>- Some of the header text is not visible, and does not show on hovering over the header<br>- Most of the content text disappears beyond the text boxes, thus hovering has to be done to be able to view text.<br>- Colour coding of the database's Matching overview is not clear<br>- No process descriptions for any of the biorefinery technologies<br>- No download functionality on database, hence no offline use of content possible<br>- No environmental impacts provided for the biorefinery technologies	- Database could be expanded to include the other remaining biorefinery technologies not included in this database	- The lack of instructions on the database webpage on how to use the database may reduce the number of users<br>- Lack of clear explanations of some of the database features like the colour coding on Matching overview will potentially discourage use of the database<br>- Database textbox design with disappearing text beyond the textbox will also potentially discourage database use.	https://magicmatch.wenr.wur.nl/

3	Charchive	Archive of (bio)char	- Access not granted at the time of the database assessment	- Access is only through access account granted by database owners after requesting for it (no access without user account)	- Database for researchers to find and share information on different biochar materials	- No update frequency provided	- Biochar products as well as the feedstock material, production and storage conditions for various samples<br>- The database description implies it contains 1 out of the 17 identified biorefinery technologies	- No openness for new content contributions	- Database is not usable without an account<br>- Requires user to be granted an account by database owners in order to access the database. User cannot create their own account<br>- Requested user account not granted yet<br>- Limited range of biorefinery products<br>- No database update information<br>- Data on process descriptions not available before access account<br>- No information on database download functionality for offline use of content<br>- No environmental impacts information provided for the biorefinery technologies	- Expanding the database content to include other biomass feedstocks and products<br>- Allowing access to the database without need for an access account<br>- Database could be expanded to include the other biorefinery technologies not included in this database	- Reduced number of database users due to the required extra step of securing an access account, hence database risks being idle and unused<br>- Reduced opportunity for receiving feedback for database improvement	https://www.charchive.org/
4	POWER4BIO	Catalogue of bioeconomy solutions	- Ease to use<br>- Database layout design is clear, simple and summarised, thus quite easy to understand.<br>- Database has links to subsections of the database for more content<br>- Database content can be downloaded for offline use	- Free to access<br>- Accessible online	-Researchers, policy makers, industry or other parties interested in bioeconomy.	- Last updated 2022	- Database contains all 10 out of the 17 identified main biorefinery technologies<br>- Database contains environmental impacts for the biorefinery technologies provided	- The database is open to new contributions	- Side products not listed against some of the products of the biorefinery<br>- Biomass feedstock is just partially characterised, and some of the feedstock is not characterised	- Database could be expanded to include the other biorefinery technologies not included in this database	- Database may have less number of users due to the partial characterisation of the biomass feedstock, in favour of databases with more complete characterisation, as most of the information will be in one place.	https://www.bio-based-solutions.eu/#/?query=%7B%22feedstocks%22%3A%5B16,132,109%5D%7D

CATEGORY III DATABASE: BIOREFINERY WIKI

No.	Database	Database content	Strengths						Weaknesses	Opportunities	Threats	Database URL
			Usability	Accessibility	Applicability	Update frequency	Scope	Openness to new contributions				
1	Tech4Biowaste	"Wiki" (similar to Wikipedia) for biowaste technologies	- Database is ease to use<br>- Hierarchichal menu design<br>- Navigation is straightforward<br>- Summary homepage provides the links to all the other sections of the database<br>- Database provides catagorisation of biorefinery technologies into pre-processing, conversion and post-processing	- Free to access<br>- Accessible online	- Stakeholders needing general information on biorefinery technologies	- Content is currently being added to the database as the database project is currently running from 2021 to 2023	- Database contains established as well as emerging biorefinery technologies to convert food processing waste, organic municipal waste, and garden waste into valuable products<br>- Covers biorefineries in Technology Readiness Level / TRL 4–9<br>- Database contains 12 of the identified biorefinery technologies	- The database is open to new contributions	- Chemical processing biorefinery conversion technologies does not include hydrolysis and oleochemical processing<br>- References to Wikipedia	- Inclusion of the other biorefinery technologies and thus increase the scope of the database	- Some of the references given in the database are taken from Wikipedia, which is not considered as a trusted/peer-reviewed source of scientific information	https://tech4biowaste.eu/database/
2	TKI · BBE	Descriptions of biogenic conversion technologies	- Database fairly easy to use<br>- Hierarchichal menu design<br>- Summary homepage provides the links to all the other sections of the database	- Free to access<br>- Accessible online	-Researchers, policy makers, industry or other parties interested in bioeconomy.	- Last updated 2020	- Database covers biochemical, thermochemical, chemical and mechanical conversion processes<br>- Database contains 6 of the 17 identified biorefinery conversion technologies	- Database is final, no further contributions	- Database is final and hence no longer being updated	- Database update with developments in biorefinery technologies<br>- Database scope can be exapanded to include biorefinery technologies not currently covered	- Database risks being obsolete due to not being updated with the latest developments in biorefinery conversion technologies	https://www.biobasedeconomy.nl/wp-content/uploads/2020/07/Biogenic-Conversion-Technologies.html#midway-1.1%20Hydrothermal%20carbonization%20(HTC)-anchor

CATEGORY IVa DATABASE: BIOMASS FEEDSTOCK GEOGRAPHICAL REGISTER

No.	Database	Database content	Strengths						Weaknesses	Opportunities	Threats	Database URL
			Usability	Accessibility	Applicability	Update frequency	Scope	Openness to new contributions				
1	S2BIOM (III)	- Biomass supply (Europe)<br>- Biomass cost/supply (Europe)<br>- Biomass cost-supply (Imports)<br>- Logistical components<br>- Value chain sustainability	- Ease to use and navigate through database<br>- Links to related databases are provided	- Free to access, with no requirement for setting up an account<br>- Database is online, hence accessible from anywhere within the set geographical regions	- Researchers, investors, governments, policymakers	- Last updated 1 November 2016	- Biomass supply (Europe)<br>- Biomass cost/supply (Europe)<br>- Biomass cost-supply (Imports)<br>- Logistical components<br>- Value chain sustainability	- This is a completed and closed project hence no further consideration for new content contributions	- Lack of regular updates to the database information, beyond the information provided for 2012, 2020 and 2030	- There is need for regular updates to the database information	- Database information may become obsolete if not updated regularly	https://s2biom.wenr.wur.nl/web/guest/conversion?p_p_id=conversions_WAR_Conversionsportlet&p_p_lifecycle=0&p_p_state=normal&p_p_mode=view&p_p_col_id=column-1&p_p_col_pos=1&p_p_col_count=2&_conversions_WAR_Conversionsportlet_cur=1&_conversions_WAR_Conversionsportlet_delta=75&_conversions_WAR_Conversionsportlet_keywords=&_conversions_WAR_Conversionsportlet_advancedSearch=false&_conversions_WAR_Conversionsportlet_andOperator=true&_conversions_WAR_Conversionsportlet_orderByCol=mainCat&_conversions_WAR_Conversionsportlet_orderByType=asc

2	ReSourcer	Norwegian bioresources	- Ease to use and navigate through database	- Free to access<br>- Accessible online	- Researchers, investors, policymakers	- No update information	- Database connects production companies that are sitting on leftover biological raw material with companies that can use these resources as input factors in new production	- Database is open to new contributions	- Database is in Norwegian and not English, thus the use of Google Translate to Translate content to English may result in loss of some of the information<br>- Content currently most useful in Norway only	- Creating a second version of the database in English, which is the language of publication for most scientific content	- Database has wider use only in Norway due to the focus being on Norway, and the language being Norwegian	https://www.resourcer.bio/

CATEGORY IVb DATABASE: BIOREFINERY GEOGRAPHICAL REGISTER

No.	Database	Database content	Strengths						Weaknesses	Opportunities	Threats	Database URL
			**Usability**	**Accessibility**	**Applicability**	**Update frequency**	**Scope**	**Openness to new contributions**				
1	DataM	Chemical and material biorefineries in the EU	- Database is easy to use<br>- Database has dropdown menus which are straightforward to navigate	- Free to access<br>- Accessible online	- Researchers, policymakers	- Last updated 10 October 2022	- Distribution of biorefineries according to the pathway for the production of chemicals or materials	- Database is open to new contributions	- Lack of detail on technology readiness level (TRL) for the reported biorefineries<br>- No process descriptions	- Inclusion of technology readiness level<br>- Inclusion of process descriptions	- Absence of the TRL information makes it a challenge for policymakers to make decisions on biorefineries<br>- Lack of process description information reduces the usage rate of the database especially for researchers.	https://datam.jrc.ec.europa.eu/datam/mashup/CHEMICAL_BIOREFINERIES_EU/index.html
2	Pilots4U	Asset register of biorefineries across Europe	- Database is easy to use<br>- Database has dropdown menus which are straightforward to navigate	- Free to access<br>- Accessible online	- Database designed for innovators, companies and research institutions operating in the bio-economy sector to gain easier access to testing facilities and scale-up and commercialise their technology faster and cheaper	- Last updated August 2019	- Database maps existing open access pilot and demo-infrastructures in Europe, creating a visible and accessible network for the European bio-economy	- Database is open to new contributions	- Lack of detail on technology readiness level for the reported biorefineries<br>- No process descriptions	- Inclusion of technology readiness level<br>- Inclusion of process descriptions	- Absence of the TRL information makes it a challenge for innovators to make decisions on biorefineries<br>- Lack of process description information reduces the usage rate of the database especially for researchers.	https://biopilots4u.eu/database

3	BEST	Facilities for the production of advanced liquid and gaseous biofuels for transport	- Database easy to navigate<br>- Database content can be filtered according to technology readiness level<br>- Database contains process descriptions<br>- Links to related databases are provided	- Free to access<br>- Accessible online	- Researchers, investors	- No update information	- Database contaimts information on facilities for the production of advanced biofuels for transport (liquid or gaseous)	- Database is open to new contributions	- No update information	- Inclusion of database update information	- Lack of database update information can result in less usage of the information as there would be no confirmation of whether the content is up to date or not	https://demoplants.best-research.eu/
4	EERE	Integrated Biorefineries in the United States of America	- Database is easy to use<br>- Database has dropdown menus which are straightforward to navigate<br>- Database content can be filtered according to technology readiness level	- Free to access<br>- Accessible online	- Researchers, investors	- No update information	- Database highlights competitively awarded bioenergy projects co-funded by the U.S. Department of Energy Bioenergy Technologies Office (BETO) Systems Development and Integration program.	- This is a U.S government controlled database, hence only updated by the state.	- Database only contains information for American facilities, hence most useful only in the United States of America<br>-Expansion of database content to include non-American facilities is not possible since this is a government database	- Inclusion of process description	- Database caters for the American market, and therefore not very useful outside of America	https://www.energy.gov/eere/bioenergy/integrated-biorefineries
